# Distinguishing between Wheat Grains Infested by Four *Fusarium* Species by Measuring with a Low-Cost Electronic Nose

**DOI:** 10.3390/s24134312

**Published:** 2024-07-02

**Authors:** Piotr Borowik, Miłosz Tkaczyk, Przemysław Pluta, Adam Okorski, Marcin Stocki, Rafał Tarakowski, Tomasz Oszako

**Affiliations:** 1 Faculty of Physics, Warsaw University of Technology, Ul. Koszykowa 75, 00-662 Warszawa, Poland; rafal.tarakowski@pw.edu.pl; 2 Forest Protection Department, Forest Research Institute, Ul. Braci Leśnej 3, 05-090 Sękocin Stary, Poland; m.tkaczyk@ibles.waw.pl (M.T.); t.oszako@ibles.waw.pl (T.O.); 3 Forestry Students’ Scientific Association, Forest Department, Warsaw University of Life Sciences, Nowoursynowska 166, 02-787 Warszawa, Poland; s211238@sggw.edu.pl; 4 Department of Entomology, Phytopathology and Molecular Diagnostics, Faculty of Agriculture and Forestry, University of Warmia and Mazury in Olsztyn, Pl. Łódzki 5, 10-727 Olsztyn, Poland; adam.okorski@uwm.edu.pl; 5 Institute of Forest Sciences, Faculty of Civil Engineering and Environmental Sciences, Białystok University of Technology, Ul. Wiejska 45E, 15-351 Białystok, Poland; m.stocki@pb.edu.pl

**Keywords:** gas sensor, application of e-nose, *Fusarium avenaceum*, *Fusarium langsethiae*, *Fusarium poae*, *Fusarium sporotrichioides*

## Abstract

An electronic device based on the detection of volatile substances was developed in response to the need to distinguish between fungal infestations in food and was applied to wheat grains. The most common pathogens belong to the fungi of the genus *Fusarium*: *F. avenaceum*, *F. langsethiae*, *F. poae*, and *F. sporotrichioides*. The electronic nose prototype is a low-cost device based on commercially available TGS series sensors from Figaro Corp. Two types of gas sensors that respond to the perturbation are used to collect signals useful for discriminating between the samples under study. First, an electronic nose detects the transient response of the sensors to a change in operating conditions from clean air to the presence of the gas being measured. A simple gas chamber was used to create a sudden change in gas composition near the sensors. An inexpensive pneumatic system consisting of a pump and a carbon filter was used to supply the system with clean air. It was also used to clean the sensors between measurement cycles. The second function of the electronic nose is to detect the response of the sensor to temperature disturbances of the sensor heater in the presence of the gas to be measured. It has been shown that features extracted from the transient response of the sensor to perturbations by modulating the temperature of the sensor heater resulted in better classification performance than when the machine learning model was built from features extracted from the response of the sensor in the gas adsorption phase. By combining features from both phases of the sensor response, a further improvement in classification performance was achieved. The E-nose enabled the differentiation of *F. poae* from the other fungal species tested with excellent performance. The overall classification rate using the Support Vector Machine model reached 70 per cent between the four fungal categories tested.

## 1. Introduction

The opening of borders for food exports from the East is worrying European consumers. The reason for this is the fear of contamination with pesticides and biological micro-organisms, especially as warfare is not conducive to the proper storage of grain seeds. Poland, for example, has received millions of tonnes of grain that has not been tested for chemical or fungal contamination because it has been classified as technical grain, which is not subject to quality standards. In theory, this grain should be used as bio-fuel, but in practice, it ends up illegally in grain mills. *Fusarium* head blight (FHB) is known to be one of the most serious diseases affecting wheat and barley worldwide. It is caused by *Fusarium graminearum* together with *F. culmorum*, *F. avenaceum* and other related fungi. Unfortunately, these fungi produce mycotoxins, but the disease itself also leads to yield losses, which is now a serious problem for food security. Pathogens can develop very quickly and in large numbers, depending on weather conditions that are beyond our control. Therefore, food security in a particular region is like a game of roulette, as we never know how heavily colonized a maize or cereal crop will be.

Fungal spores spread through the air over a wider area, so localized residues under cereal crops (i.e., from the previous harvest) are less important, especially in regions where the spores are likely to be spread by the wind. As the fungi grow, they produce mycotoxins that are water-soluble and can, therefore, be washed out of the infected tissue.

This situation makes it urgent to look for methods to monitor the presence of harmful fungi in stored grain [1]. This is all the more true as important epidemiological issues have recently emerged, including an apparent shift in the prevalence of *Fusarium* species on infected heads in Europe towards *F. graminearum* and the presence of multiple chemotypes and variants of aggressiveness within the species in a given region [2]. The host is most susceptible to infection during and shortly after flowering. A warm, humid environment characterized by frequent rainfall or heavy dew is highly favorable for fungal growth, infection, and disease development in the head tissue. In storage facilities, the rate of fungal colonization of the seeds is also determined by the prevailing humidity and temperature. However, under wartime harvesting conditions, the seeds may be insufficiently dried and of poor quality and still be shipped to Europe to buy the necessary armaments.

Differentiating between different types of *Fusarium* fungi is possible using various diagnostic methods. They consist of the classical cultivation method, immunological and genetic tests based on the PCR reaction, and direct DNA sequencing [3].

The advantages of the molecular detection of fungal contamination are high accuracy, repeatability, sensitivity at the level of pg, and even fg of DNA [4]. Unfortunately, these methods are quite expensive and are generally only suitable for laboratory use as they require a high degree of cleanliness. There are other disadvantages of the genetic methods of fungal spoilage detection. For the measured example, the sample must contain the DNA of fungi. In the case of stored grain, the infected material can be deeply buried, and it is not obvious that the collected sample will contain such material.

The presented research approach relies on the detection analysis of volatiles emitted by the fungal spoilage. Even if the infected grain is covered by layers of healthy grain, the volatiles diffuse and may be detected by various methods.

In general, fungi produce a variety of volatile substances. They are often species-specific, which enables us to recognize and distinguish them from each other. Many insects and other animals recognize certain types of fungi by their smell. In one such situation, an attempt was made to use odors and compounds to distinguish between different species of the genus *Fusarium* that infest wheat grains.

Various techniques for the chemical analysis of gases can be used to detect and distinguish different odors. The most effective is the use of classical chemical analysis methods, such as gas chromatography in conjunction with a mass spectrometer, which can be used to determine the individual chemical components and their relative concentrations. In practice, this method is limited to applications under laboratory conditions.

Another approach is the use of an electronic nose (e-nose) [5,6,7], which does not identify the individual chemical components of the measured gas mixture but uses a series of non-specific gas sensors and pattern recognition techniques. There are currently several commercial electronic noses on the market. However, there are numerous research reports on the construction of low-cost devices that could be sufficient for many applications. One of the necessary decisions in the search for home-built devices is determining the choice of gas sensors. In such a case, the MOX (metal oxide sensors) available on the commercial market are often the preferred choice [8]. At this point, it should be noted that the application of such sensors in electronic noses is a secondary purpose, as they are designed and developed for other applications [9].

The most common method for operating MOX sensors in e-noses is to examine the pattern of the sensor’s response to changes in the composition of the measured gas. In this case, a pneumatic system is required to ensure the repeatability of the measurement conditions and the speed of the gas change [10]. However, simpler solutions can also be used [11]. Most commercially available sensors are designed for this type of operation. Another method is to analyze the response patterns of the sensor to the change in operating temperature in the presence of the measured gas.

Balasubramanian et al. [12] evaluated an artificial olfactory system for grain quality discrimination. Gobbi et al. [13] demonstrated that electronic noses can predict high and low fumonisin contamination in maize cultures. Mota et al. [14] have investigated the identification of fungal species using electronic noses. There are reports on the use of electronic noses to study the odor of various cereal grains infested by fungi [15,16], oilseed rape [17,18] and rice samples [19,20]. There are also numerous research papers focusing in particular on *Fusarium* species: Falasconi et al. [21] for *Fusarium verticillioides* in maize; Presicce et al. [16] for *F. poae* in wheat; Perkowski [22] for *F. culmorum* in wheat and triticale cereals; Eifler et al. [23] for *F. graminearum* and *F. culmorum* in wheat grains; Nordstorm et al. [24] for *F. circinatum*; Feng et al. [25] for *F. oxysporum* in tomato processing; Labanska et al. [26,27] for basal rot infections in onions and shallots and Camardo Leggieri et al. [28] for *F. graminearum* mycotoxins in wheat. In our recent studies [29,30], we also analysed the contamination by *F. culmorum*, *F. graminearum* and *F. oxysporum*. Sennik et al. [31] reported experimental results on differentiating between healthy wheat plants and plants infected with Fusarium head blight based on sensing the ambient gases in the plant environment using a gravimetric electronic nose enabled by a functionalized capacitive micromachined ultrasonic transducer array and machine learning algorithms. Cui et al. [32] presented predictive models for assessing the risk of *Fusarium pseudograminearum* mycotoxin contamination in post-harvest wheat with multi-parameter integrated sensors. Ray et al. [33] reported a non-destructive asymptomatic early disease prediction method employing ROS-induced differential volatile emissions from dry rot-infected potatoes. An evaluation of the performance of a metal oxide electronic nose for the detection of aflatoxin in artificially and naturally contaminated maize was reported by Machungo et al. [34]. Zhou et al. [35] demonstrated the feasibility of the detection of infested rice using an electronic nose.

We can observe increasing interest in the field of electronic nose research. New types of devices have been proposed [36,37], and advances and future perspectives regarding electronic nose technologies have been reviewed [38,39,40,41] in recent studies.

Other research has reported different emerging methods used for the detection of *Fusarium* contamination. Belyakov et al. [42] presented a method of optical diagnostics of grain seeds infected with *Fusarium*. Mustafa et al. [43] reported enhancing *Fusarium* head blight detection in wheat crops using hyperspectral indices and machine learning classifiers. Abu et al. [44] presented an electronic tongue and box-PCR for the categorization of different *Fusarium* strains. Lacastagneratte et al. [45] demonstrated the detection of fusariosis on black pepper plants using a multispectral sensor. Trakselyte-Rupsiene et al. [46] described the challenges of acoustic screening for deoxynivalenol and deoxynivalenol conjugates reduction in contaminated wheat-based products. Beilinson et al. [47] described amperometric enzyme immunoassay sensors in the determination of *Fusarium oxysporum* antigens. Chang et al. [48] reported the development of rapid detection methods for *Fusarium oysporum* f. sp. melonis in melon seeds. Liu et al. [49] explored non-destructive quality-detection techniques for cereal grains.

In this study, we report on the investigations into the use of the prototype electronic nose with the two gas detection methods mentioned above. The analysis of the collected data was performed using two common machine learning classification algorithms, with the aim of using a hybrid electronic nose method with a fusion of data collected in both the gas adsorption phase and the sensor heating modulation phase. The constructed low-cost electronic nose was used to distinguish four types of *Fusarium* fungi infecting wheat grains.

## 2. Materials and Methods

### 2.1. Sample Preparation

Four species of fungi belonging to the genus *Fusarium* have been used to infect wheat seeds: *F. avenaceum*, *F. langsethiae*, *F. poae* and *F. sporotrichioides*. All Fusarium species were inoculated onto fresh Potato Dextrose Agar—PDA medium (20 g dextrose, 15 g agar, 4 g potato starch, and 1 L distilled water) and then incubated at 22 °C in the dark until the mycelium covered the entire surface of the dish. The prepared plates served as inoculum for the inoculation of wheat seeds. The samples for analysis were prepared seven days before the start of the measurements. Fifteen grams of wheat seeds were poured into sterile Petri dishes with a diameter of 90 mm. The seeds were moistened by adding 5 mL of distilled water to each plate using a pipette. Then five pieces of agar (taken with a 5 mm diameter cork borer) were transferred to each plate in a laminar chamber together with fresh mycelium from previously prepared *Fusarium* plates.

Fungi of the genus *Fusarium* grow very quickly at room temperature on artificial media and easily infect the grain. There were no differences in the effectiveness of inoculum between different *Fusarium* species in this respect.

### 2.2. Electronic Nose Experiment

#### 2.2.1. Self-Built Electronic Nose Device

In a number of our previous publications, we have reported on the step-by-step development of our own electronic nose devices [11,30,50]. For the reader’s convenience, we would like to provide here a brief description of the current version of the device used in the experiment.

**Figure 1:**
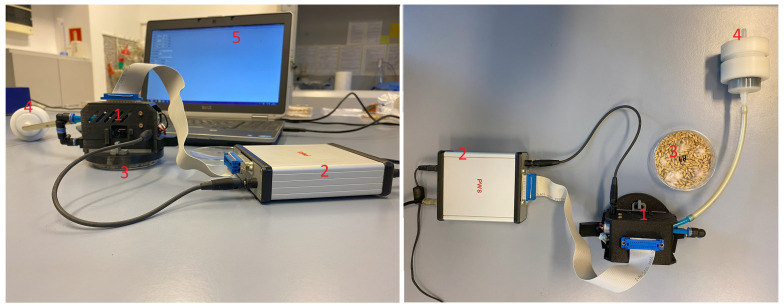
Measurement setup of the electronic nose. (1)—sensor chamber, (2)—control unit, (3)—measured sample in a Petri dish, (4)—charcoal air filter, (5)—laptop controlling the measurements.

In Figure 1 we show an experimental setup of the electronic nose with a sample contained in a Petri dish. The constructed device contained a set of six commercially available metal oxide (MOX) gas sensors from Figaro Inc. (Osaka, Japan) [9]. The list of sensors with their target gases can be found in Table 1. The sensors are mounted in a sensor chamber [11], which can be placed on the sample to be measured and exposes the sensors to the volatile gases present there. The sensor chamber can be closed and then clean air is pumped in to clean the sensors or to record the base signal. The sensors are connected to the electronic control unit [50], which is used to measure the sensor resistance. MOX sensors must be heated to a temperature of several hundred degrees Celsius for their operation, which is achieved by an internal heater supplied with external voltage. The control unit used in our design makes it possible to modulate the heating voltage of the sensors and to change the response of the sensors depending on the disturbances generated by this method [29,50].

The electronic nose device we use in our research is one of the low-cost devices. The electronic circuit diagram of the device used is similar to the one in Ref. [50]; the differences lie in the number of mounted sensors. In addition to the sensors and other electronic circuit elements, the cost of the constructed device consists of the self-printed 3D sensor chamber, the self-made carbon filter and the pneumatic pump. We estimate the total cost of the components for assembling the device to be about EUR 450 (not including labor costs), which depends on the current supply of the individual components prices (Table 2).

#### 2.2.2. Output Signals of the Electronic Nose

**Figure 2:**
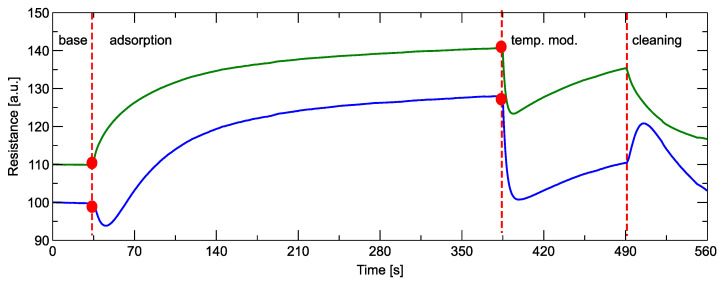
Examples of shapes of sensor electronic nose response during one measurement cycle of a sample. (blue)—the shape of the response of the TGS 2602 sensor, (green)—the shape of the response of all other types of used sensors (Table 1). Various stages of the measurement cycle are indicated inside the figure. The red dot at the beginning of the gas adsorption phase and at the beginning of the sensor temperature modulation phase represent the baseline response level, which is different for each of the response phases.

The signals that allow the electronic nose to distinguish between different odors should be measured as the sensor’s response to perturbation [51]. Our device uses two types of perturbation. The first, which we used, is to measure the sensor’s response to the change in sensor conditions between the clean air and the measured odor in which the sensors are immersed. By opening and closing the sensor chamber, these conditions can change abruptly as the odor fills the space near the sensors. The dynamic response, i.e., the change in sensor resistance, is recorded. The second type of disturbance is a change in the temperature of the sensor heater by modulating the voltage of the sensor heater. In the present studies, we applied an abrupt drop of the voltage by a small percentage from the nominal value of 5 V [9] in a single step [50]. In Figure 2, we show a schematic representation of the sensor response during a measurement cycle of an odor sample.

The first 35 s of the measurement were performed under clean air conditions, and the baseline level was recorded. Then, the shutter of the sensor chamber was opened, and the reaction during the adsorption phase was measured. The measured adsorption phase lasted 280 s, as this time was sufficient to reach the quasi-steady state of the sensor response. Two types of sensor behavior were observed during this phase. For the TGS 2602 sensor, an abrupt and short drop in response was observed immediately after exposure to the measured gas, and after reaching the minimum, an increase in the measured conductance was observed, which was comparable to the response of all other sensors. After reaching the stable response level, we changed the voltage of the sensor heater from a nominal 5 V to 4.75 V. This led to a decrease in the sensor temperature, and the shape of the signal response was characterized by an abrupt drop. After reaching the minimum, an increase in the measured conductance was observed. The choice of such a profile of sensor heater voltage modulation was a result of our previous research [50], in which we demonstrated that the shallow drop of the heater voltage is a preferred method for our electronic nose. We did not measure the sensor response in the heating modulation phase until the steady state was reached but closed the sensor chamber after 70 s and started sensor cleaning. The sensor response was recorded for a while, but this signal was not used for the analysis. During the sensor cleaning phase, we also increased the temperature of the sensor heater (not shown in Figure 2) to prepare the sensor for the subsequent measurement of the next sample.

When we analyzed the data after the experiment, we found that the TGS 2603 sensor behaved differently from the other sensors. Although this sensor responded to the measured samples, its time for cleaning and restoring a stable state under clean air conditions was much longer than that of the other sensors used in our electronic nose. This was particularly observed when measuring samples of *F. poae*. The recovery time was longer than assumed in our experiment, and the state of the sensor in subsequent measurements still “remembered” the previous measurement. For this reason, we decided to exclude this particular sensor from the analysis presented in this report. For all other sensors, the applied sensor cleaning time was sufficient, which we could determine by checking the overall sensor recovery curve.

#### 2.2.3. Measurement Series with the Electronic Nose

We performed the measurements of the samples over six consecutive days, using two Petri dishes each day with each variant of the infected wheat grain. The measurements were carried out in a random order, with the random number generator from an Excel spreadsheet determining the order of the samples for each day of the experiment. In total we collected 14–15 observations for each variant. A few observations were removed from the analysis as they were found to be corrupted. In total 60 observations were collected.

The Petri dishes used were kept closed between measurements. The electronic nose sensors were connected to the power supply throughout the experiment and kept outside when not used for measurement. According to the manufacturer’s recommendations, the sensors were pre-warmed for one week before the experiment. During that time the sensors were kept in clear air conditions which allowed them to be cleaned of previous odor residue. We decided that the duration of the experiment of five days was short enough to assume the stability of the sensor response and that there was no need for any kind of recalibration during that period.

The experiment was conducted under controlled conditions with a constant temperature of 21 °C and a humidity of 30%. The measurements were carried out in a laminar flow cabinet (Telstar, Bio II Advance, Barcelona, Spain), and the measured samples were stored there for the entire week of the experiment.

### 2.3. Machine Learning Modeling for Classification

#### 2.3.1. Extraction of Predictors

The entire sensor curves can be represented by a smaller number of features that describe their shape and should contain the most important information needed to discriminate between the measured samples. Different methods have been proposed [52] to define a set of features that can be useful for applications in the field of electronics research.

When looking at the shapes of the sensors’ response curves (Figure 2), we notice some patterns that help us define what kinds of features can be helpful to describe the curve. What is important to notice is that the sensor response’s absolute value is not used for the analysis. According to the manufacturer’s recommendation [9], the meaningful property is the sensor response relative to the response level in the presence of clean air $G/G_{0}$. It should also be noted that this reference level for the phase of the modulation of the sensor temperature is chosen differently, as the stable state of the response before modulation. This is schematically represented in Figure 2.

The list of features extracted from the sensor response curve with a short description can be found in Table 3. The proposed features are based on the global features of the whole curve of the sensor response during the considered phase (e.g., final equilibrium state, the area under the curve corresponding to the average response), but we also made an effort to capture the response behavior at the beginning of the perturbation (switching on the gas or changing the sensor temperature), in agreement with our previous experiments [29,30], as this region of the response contains useful information for discriminating between different odors.

The chosen predictors allow us to reduce the dimensionality of the problem. The choice of the features was based on the examination of the sensor response curves, allowing for the extraction of their main features, which could be sufficient to reconstruct the main behavior of the response with the reduction of noise. More sophisticated features could be extracted and multiple studies have focused on that issue, demonstrating possible improvements in the performance of the classification models in such cases. Some authors proposed an advanced deep learning approach allowing for the automatic discovery of the predictive features extracted from the gas sensors. However, in our opinion, that is beyond the intended scope of the presented research. In our research, we deliberately chose the basic features, which do not capture fine changes in the sensor response but only their overall characteristics. In our opinion, the limited number of collected observations only allows for such an approach to be suitable if we would like to avoid reporting some artifacts arising from statistical data fluctuations.

#### 2.3.2. Machine Learning Models

In order to estimate the possibility of discrimination between the studied categories of samples based on the signals collected by the electronic nose measurements, we trained two machine learning classification models using (i) Logistic Regression and (ii) Support Vector Machines with linear kernel algorithms. That are ones of the most commonly used techniques in the field of classification. We have selected these techniques from the large number of different methods proposed because we have to admit that we have only collected a rather limited number of experimental observations. The electronic nose measurements, especially for biological samples, are characterized by a relatively high noise compared to the signal. For this reason, we have come to the conclusion that the preferred machine learning algorithm should be characterized by a not very flexible decision boundary between separated regions. This should limit the possibility of finding very fine patterns in the data space, but thereby also limit the possibility of over-fitting and training patterns arising from statistical fluctuations in the data distribution.

Three models were trained and evaluated using the collected data. The first one is used as a predictor of a subset of features, extracted from the adsorption phase of the sensor response. The second model is used as a predictor of a subset of features extracted from the sensor response during the temperature modulation phase. The third model used all features extracted from the whole response curve (Table 3).

#### 2.3.3. Metrics of Model Performance

Three commonly used metrics of model classification performance were used in our modeling: (i) Accuracy, defined as the proportion of correctly classified cases out of the total number of cases; (ii) Precision, defined for each classified category of observations as the ratio between the number of correctly classified observations and the number of observations classified by the model as belonging to that particular category. The precision metric focuses on the confidence that the classified dataset truly belongs to a category of interest; (iii) Recall is defined as the ratio between the number of correctly classified cases from a category of interest relative to the total number of cases in that category. The recall metrics focus on the ability to recognize observations from a particular category and are not penalized for observations from other categories that were incorrectly classified as belonging to that category.

The machine learning classification model is useful if it can be applied for scoring new data, not used for the model training. If the model performance is assessed using the training data it is largely overestimated as the model may fit to statistical fluctuations in the data, and not only to the patterns useful for discrimination between the treatments. The common practice is splitting the dataset to the training and testing parts, and then using the former for model training and the latter for the performance assessment. In the case of small size datasets, more sophisticated techniques are commonly used, which are based on cross-validation methods [53].

To honestly assess the performance of the model, we applied a leave-one-out cross-validation technique. In a loop over all collected observations, we trained a model with all collected observations except the excluded observation and estimated the performance metrics of the model using this one observation. At the end of the loop, the average value of the model performance was reported. The pipeline for training and testing the model also included a default scaling of the training dataset, which prevents information from the test observations from entering the training set.

To evaluate the confidence of the calculated performance metrics we applied the bootstrap method [54,55] with 1000 repetitions. The 95% confidence intervals were calculated. It should be noted that such calculations are very computing-intensive, since the model training needs to be repeated in a bootstrap loop as well as in the cross-validation loop.

#### 2.3.4. Software Packages

All data processing was performed with Python 3.10 codes, using the scikit-learn package [53] for model training and performance evaluation.

## 3. Results and Discussion

### 3.1. Distribution of the Measurement Data

The data collected by the electronic nose (Figure 2) and also the features extracted from the sensor response curves (Table 3) are multidimensional data, which makes them difficult to visualize in order to gain insight into the patterns in the data that could be useful for distinguishing between the different categories of samples studied. A helpful approach is to transform the data into lower dimensions. Our choice fell on the application of linear discriminant analysis (LDA). Our goal was to choose a data projection technique to lower dimensional space that emphasizes differences between the studied categories of data. We chose a linear method of transformation as the machine learning model used in our research also belongs to the category of linear models. That facilitates a comparison and interpretation of the similarities in the patterns in data. To avoid any confusion, we would like to note here that the machine learning classification models were trained using all features.

**Figure 3:**
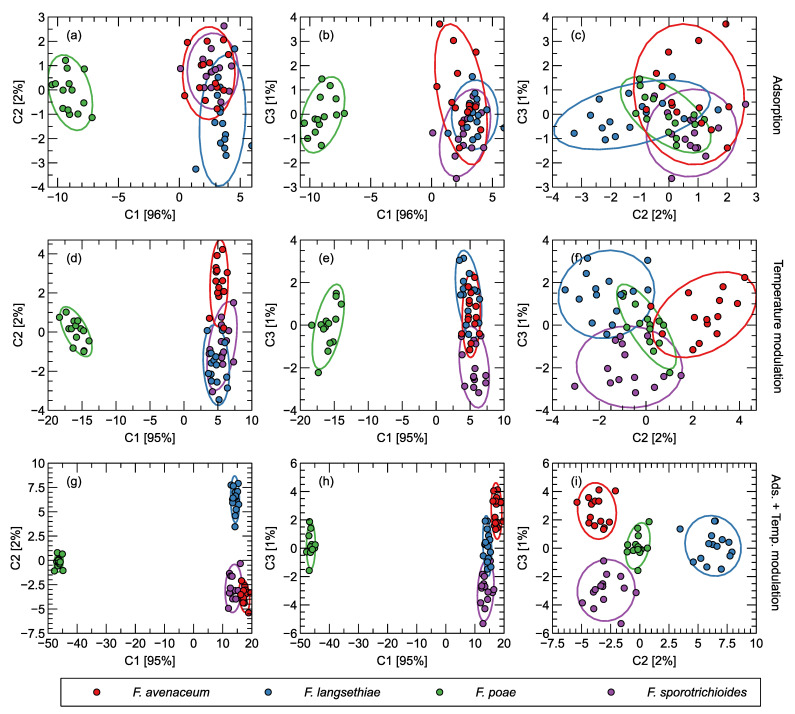
(**a**–**i**) Distribution of observations obtained by electronic nose measurements as LDA projection of features extracted from sensor response curves. The rows of the sub-figures represent different sets of features: extracted from the adsorption phase, extracted from the temperature modulation phase, and extracted from both phases of the response, as indicated on the right side of the figure. The columns of the sub-figures represent different projections onto the LDA components c1–c2, c1–c3, and c2–c3, as indicated at the top of the figure and in the axis labels. The percentage of variance explained by the selected components is indicated in the axis labels. Confidence ellipses for two standard deviations are shown.

In Figure 3, we show the data projection on two dimensions generated by the LDA transformation. We compare the three cases where different features were extracted from the collected data: (a) the adsorption phase of the sensor response, (b) the temperature modulation phase of the sensor heating, (c) the collection of features from both phases of the sensor response.

As can be seen in Figure 3, there is a clear separation in the observations of the measurements of samples infested with *F. poae* compared to three other analyzed species in all cases considered. This could indicate that the differentiation of *F. poae* from other species can be achieved by applying the analysis of the signals collected by the electronic nose.

If we look at the data points representing experimental observations obtained from the measurements of three other samples, we can see that they represent a single cluster, especially in the case where the features are extracted from the adsorption phase of the sensor response (Figure 3a). One can notice here that the observations representing samples of *F. langsethiae* are pushed out of the cluster of data representing *F. avenaceum* and *F. sporotrichioides*. Nevertheless, about half of the observations still overlap with the clusters formed by the first two fungal observations. For the features extracted from the temperature modulation phase of sensor heating (Figure 3d), the differentiation seems to be better, but in this case, a clear cluster is formed by observations representing *F. avenaceum*. A clear separation of the cluster formed by observations representing *F. langsethiae* can be observed for the case where the features were extracted from both phases of the sensor response (Figure 3g).

When we look at the patterns that appear in the visualization in Figure 3, we should also look at the proportion of variance in the dataset that is explained by each of the components of the LDA projection. As we can see, the first component captures 95–97% of the total variance. The pattern observed in the differentiation between the samples of *F. poae* and other species is due to the difference in the first LDA component, so we can expect this pattern to be the strongest found in the dataset. The patterns found in the data distribution that allow us to distinguish between the samples of the other three species are found when we look at the second and third LDA components, which capture much less variance in the data set and are therefore much weaker. Because the second and third LDA components capture such a small proportion of the total variance, we should be cautious as these patterns may be caused by noise and statistical fluctuations and do not represent the signal differences that could be used in practice to discriminate between the species studied.

A reader should be warned to treat the LDA results cautiously, especially in the case of analysis when the features extracted from both phases of (A) adsorption and (T) temperature modulation of the sensor response are merged. As we noted, 60 observations were used in the analysis. In the A subset, there were 31 features, and in the T subset there were 45 features, which gives a total of 76 features in the AT subset. The LDA analysis should be viewed as giving an intuitive explanation of the emerging patterns, and basically, the same patterns were found in the A, T, and AT subsets of features.

### 3.2. Classification Performance through Machine Learning

As we noted in the previous section, it is important to verify whether the data patterns observed in the visualizations can be useful for applications to distinguish between the *Fusarium* species. Such verification can be performed by training a machine learning model for classification and testing its performance on an independent subset of data that was not used to train the model.

**Figure 4:**
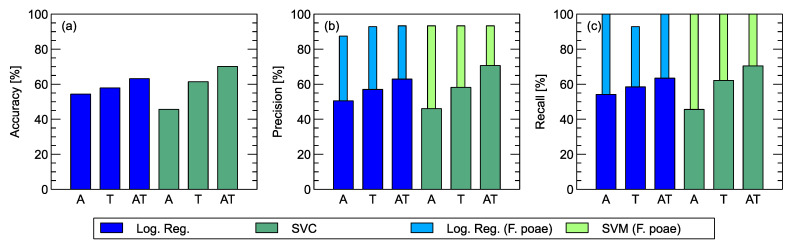
Measures of the performance of classification models obtained by Logistic Regression and Support Vector Machine algorithms estimated with the leave-one-out cross-validation method. (**a**) Accuracy, (**b**) Precision, (**c**) Recall, as indicated in y-axis captions. Comparison of different sets of predictors extracted from the adsorption phase (A), the temperature modulation phase (T), both phases of the sensor response (AT). For Precision and Recall, the average of the performance for all species is compared with the performance of *F. poae* alone. The numerical values of the metrics with corresponding confidence intervals are presented in Table 4.

In Figure 4a, we can compare the prediction accuracy of the two types of machine learning classification models for three considered sets of features extracted from the sensors’ response curves and used as predictors. As we can see, there is a trend in both cases that the classification accuracy improves when we use the data collected in the temperature modulation phase of sensor heating compared to the case where the features were extracted from the data collected in the gas adsorption phase. Further improvements in classification performance are also observed when the features extracted from both phases of the sensor response are used as predictors. These trends are consistent with the patterns found when examining the LDA plots, as described in the previous section.

A look at the results of the trends in the Precision and Recall metrics provides further insights. If we compare the differences between the three groups of predictors analyzed, we can see a trend toward improvement. Let us first focus on the Recall metrics (Figure 3c). As we can see, the hit rate of *F. poae* is 100% in the case of SVM classification and is equally high in the three cases considered. This means that all observations of *F. poae* were correctly classified by the model. An improvement in the average Recall, in this case, means that there is an improvement in the discrimination between the three remaining species of *Fusarium*. The trend visible in Figure 3b is also worth mentioning. As can be observed, the Precision of *F. poae* is constant for the three cases analyzed but is below 100%. This means that some observations of other species were misclassified as *F. poae*, but there was no improvement in the classification of these particular observations when different sets of predictors were used. The overall trend in average Precision was then caused by better classification between the three other species of *Fusarium* used in the experiment. We found a similar pattern when we examined and discussed the LDA visualizations, but this was not confirmed by the test on an independent subset of data that we performed in the case of machine learning modeling.

### 3.3. Comparison with Other Results of Electronic Nose Measurements

The results of electronic nose measurements are difficult to compare to other research. Various fungi species were used in experiments, but since there is no objective measure or definition of odor, the published results report classification between various sample categories. That makes it often possible to compare only in the context of the published research.

In the previous research [56], we reported the results of a very similar experiment, using the same species of *Fusarium*, but the measurements were performed using a state-of-the-art commercial electronic nose device, namely PEN3. The obtained accuracy in both cases was very similar—in both cases it reached 70%. Also, very similar patterns concerning the ability to differentiate between *F. poae* and other species were found. There are clear advantages of the commercial electronic nose, such as automatics and ease of use or a much shorter time of a single measurement. During the same time, we were able to collect two times more experimental observations using PEN3 than using our low-cost device. The reduction in the time of measurements was caused by two factors. First of all, in our construction, we used two types of sensor responses—adsorption, and temperature modulation phases. In addition, the PEN3 is equipped with a more sophisticated and faster sensor cleaning procedure. We are convinced that the overall results certify that self-constructed electronic nose devices may be an interesting and affordable alternative for some applications.

In many studies, the reported performance of electronic noses is much higher for the case of applications of self-made as well as commercial devices. We can mention here that, in our previous research, where we were using the preceding versions of our current construction, we obtained accuracy up to 78% for differentiation between *F. oxysporum* and *Rhizoctonia solani* [57] and 97% for differentiation between *F. oxysporum* and *Phytophthora plurivora* [50]. The first of these mentioned results was obtained by the device with the sensor adsorption phase only, and the second one with the sensor heater modulation phase only.

Labanska et al. [26,27] recorded the experiments with a PEN3 e-nose device for the detection of *Fusarium* infection in onions, with a classification accuracy up to 89%. Also, Feng et al. [25] reported almost perfect detection of *F. oxysporum* infection in processing tomatoes using a low-cost electronic nose.

The accuracy obtained in the present research of differentiation between the studied *Fusarium* species of 70% may seem to be rather low, but that depends not only on the quality of the device but also on the characteristics of measured odors, which may be easy or difficult to differentiate.

### 3.4. Common Pattern Revealed by GC-MS Analysis

The patterns found in the analysis of data obtained from the electronic nose measurements can be compared to some patterns found in the analysis of the chemical composition of volatiles emitted by the studied fungi. The chemical composition of the volatiles may be analyzed using gas chromatography coupled with mass spectrometry (GC-MS). An indisputable advantage of such type of analysis is its objectivity as it allows the identification of individual components present in the sample as well as the proportions of the abundance components. Measurements of the concentration of the components are also possible but require a more advanced measurement protocol. However, the GC-MS method requires expensive equipment, uses a demanding measurement protocol, and can only be operated by experienced staff. That limits its application to laboratory usage.

**Figure A2:**
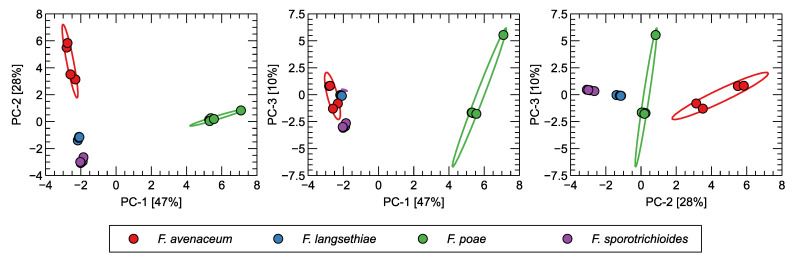
Principal Components Analysis transformation of the proportion of chemical compounds identified in the GC-MS analysis of measured samples. The sub-figure columns represent different projections onto the PCA components, as indicated in the axis labels. The variability explained by the components is indicated in the axis labels. Confidence ellipses for two standard deviations are shown.

In Figure A2 in Appendix A, we present a visualization of the Principal Component Analysis of the chemical composition detected in similar samples of wheat grain infested by the four studied species of *Fusarium* fungi. As one can notice in this figure, the volatiles of *F. poae* are distinct from other studied species, and the difference is observed along the first, the most important principal component. Differences between other studied samples are only along the second principal component, which captures much smaller variability in the data. Even if in this figure, the cluster belonging to the three considered species of samples are distinctly separated, which may be a consequence of a very small number of measured samples [51].

The pattern found in the analysis of the chemical composition of the samples of the fungi species confirms that the patterns found in the analysis of the electronic nose data are caused by different compositions of the odor profile and not by other effects.

### 3.5. Threat to Food Security from Fungi

In our opinion, it may be interesting to provide more information concerning the influence of the studied species on the environment, justifying the importance of the chosen subject of experiments.

There are more than 1.5 million species of fungi worldwide, of which pathogenic species can infect plants at various stages and cause considerable financial damage [58]. An important group of plant pathogenic fungi are the *Fusarium* species. *Fusarium* fungi colonize the soil and cause many economically important plant diseases and, therefore, became the factor of our interest and target for our study with e-nose devices. Many species of the genus *Fusarium* are not only pathogenic for plants but also cause various diseases in humans and livestock. In addition to diseases, one of the most dangerous properties of these fungi is their ability to produce dangerous secondary toxic metabolites, commonly known as mycotoxins. Some of the most important toxins produced by various *Fusarium* species are fumonisins and trichothecenes. *Fusarium* species are distributed worldwide and have a very broad host range, including many economically important crops and plant species. Most plant diseases are caused by *F. solani*, *F. oxysporum* and *F. graminearum*. *Fusarium* species can infect grains in storage, and in this case, electronic noses appear to be potentially very useful in their detection. Although plant infections occur in the fields where the fungi cause crop losses, they also infect the grains later and cause damage during their storage. The earlier they are detected, the more effective the control methods (chemical or biological) can be.

Globally, toxigenic *Fusarium* species are particularly dangerous in terms of the risk of poisoning [59]. They are widespread pathogens of wheat and other cereals. In Poland, for example, 449 wheat heads from six locations were found to be heavily colonized by pathogens of the genus *Fusarium* in the 2009 vegetation season. Primarily *F. culmorum* (72.1% on average), *F. graminearum* (13.4%) and *F. avenaceum* (12.5%) were identified, while *F. cerealis* (1.8%) and *F. tricinctum* (0.2%) were observed less frequently. Unfortunately, their presence was associated with the production of mycotoxins (deoxynivalenol, zearalenone, and moniliformin) found in the grain and chaff fractions. Moniliformin (MON) was found in all samples infected with *F. avenaceum*, while deoxynivalenol (DON) and zearalenone (ZEA) were present in grains infected with *F. culmorum* and *F. graminearum*. The highest concentration of DON was found in a grain sample from central Poland (77 µg/g), which was due to the high temperatures in July and August and the numerous rainfalls in July. Even if the E-noses are only useful for the detection of pathogens, it is somewhat simplistic to risk the possibility that the development of their populations will sooner or later lead to contamination of the grain with mycotoxins (trichothecenes, zearalenone, enniatin, and bibericin) and the resulting health problems.

### 3.6. Changes in the Occurrence of Pathogenic Fungi in Agriculture and Strategy for Their Identification

The classical methods of identifying fungal isolates are based on species-specific PCR or the sequencing of translation elongation factors. Our research aims to replace these in the future with e-nose sensors, which could represent a cheaper and faster solution. This is all the more true as considerable differences were found between the two years of the study [60], both in the frequency and in the species composition of the strains collected. A total of 107 *Fusarium* strains belonging to 10 species were obtained from the 304 ear samples analysed: *F. graminearum*, *F. culmorum*, *F. sporotrichioides*, *F. poae*, *F. avenaceum*, *F. oxysporum*, *F. verti-cillioides*, *F. equiseti*, *F. tricinctum* and *F. cerealis* were isolated in 2012 and 2013 [60]. In our studies, some of these species have already been identified by odor, and *F. poae* in particular differs from the others in this respect. In the genetic studies mentioned above, the presence of genes for the mycotoxin biosynthesis pathway was detected in large numbers, indicating the potential toxicity of the *Fusarium* fungi analysed. The early detection of pathogens by e-nose can therefore determine whether or not a seed lot is authorized for human or animal consumption.

Fusarium head blight (FHB) of cereals is an important underlying disease that has a negative impact on cereal production worldwide [61]. In 2016 and 2017, major outbreaks of FHB occurred in wheat crops in Poland, and the diversity of Fusarium head blight fungi responsible for these outbreaks was investigated. This fact alone justifies the reason for the present investigation using the e-nose. Using molecular biological methods (TaqMan assays), fungi were identified from 463 ears of wheat, among which *F. graminearum* s.s. (81%) and to a lesser extent *F. avenaceum* (15%) predominated.

In addition, changes in the fungal populations in Poland were observed, which were accompanied by the displacement of *F. culmorum* by *F. graminearum* s.s. This is probably related to the increase in maize production, which has increased significantly in Poland over the last ten years.

By analyzing the species composition in populations of *Fusarium* spp. isolated from winter wheat ears in southern Poland, the frequency of genes involved in the synthesis of mycotoxins (deoxynivalenol, nivalenol, and fumonisins) was also assessed [60]. This is of particular importance for food safety reasons.

In Poland, there are numerous examples of the identification of fungi and their toxic metabolites [62], e.g., in 78 samples of wheat (six varieties) from 13 locations in different climatic zones, ten percent of all fungal isolates obtained were *Fusarium* species. The most common were *F. poae* (64%), *F. tricinctum* (15%), *F. avenaceum* (8%) and *F. culmorum* (6%). For this reason, our first tests with the electronic nose are focussed precisely on the detection of this group of fungi. The aforementioned report on the natural contamination of wheat with *Fusarium* toxins in Poland showed, among others, the presence of deoxynivalenol in 50% of samples with the highest contamination level (xmax = 102 µg/kg), nivalenol in 30% of samples (xmax = 99 µg/kg), moniliformin in 62% of samples (xmax—495 µg/kg). These data confirm the need for new, rapid methods for the detection of fungal contaminants in agricultural products, and perhaps electronic noses will fulfill such a function in the future.

There is currently great interest in the food production chain in the development of techniques for the rapid early detection of mycotoxic fungi, e.g., molds [63]. The development of different types of sensors that respond to the presence of volatiles produced by fungi is being investigated as a potential method for their detection. Commercial ‘electronic noses’ have already been successfully investigated for the potential detection of mycotoxic molds in raw foods. There is also interest in the use of volatiles to differentiate between non-mycotoxic and mycotoxic strains of the genera *Fusarium* (section Liseola), *Aspergillus* (section Nigri) and the species *Penicillium verrucosum*. Our research using e-nose devices fits well into this trend.

It should be noted that the genetic methods of fungi detection and recognition are a gold standard for detecting and recognizing fungi and mycotoxin spoilage. A trace quantity of DNA may be detected. Also, the operation of electronic noses relies on the detection of common types of volatile chemical components, which are not specific to the fungi of interest. Specific molecules of fungi, especially mycotoxins, are not volatile. According to Cheli et al. [39], when reviewing the current status of the e-nose technology, the presented accuracy is still limited and unsuitable for in-field applications for the detection of mycotoxins. Further studies are needed in this field of research. That was a goal of our paper, which seeks for improvements in the construction of e-nose devices and data analysis methods.

## 4. Summary and Conclusions

The proposed homemade electronic nose belongs to the category of inexpensive devices. It was built using commercially available sensors of the TGS series from Figaro Corp. Two models of gas sensors are used to collect the signals useful for discriminating the analyzed samples. First, the electronic nose detects the transient response of the sensors to the change in operating conditions from clean air to the presence of the measured gas. A simple gas chamber was used to produce an abrupt change in gas composition near the sensors by opening the shutter. An inexpensive pneumatic system consisting of a pump and a homemade charcoal filter was used to supply the system with clean air, which was used to clean the sensors between measurement cycles. The second function of the electronic nose is to detect the response of the sensor to a disturbance in the temperature of the sensor heater in the presence of the measured gas.

It should be noted that the sensors used are designed to operate at a constant sensor heating temperature, as recommended by the manufacturer. However, our research shows that they can also be successfully used with temperature modulation. In our approach, we used a flat modulation of the heating voltage in a range close to the nominal value recommended by the manufacturer.

The constructed electronic nose device was used to distinguish between four categories of samples, namely wheat grains infected with four different types of *Fusarium* fungi: *F. avenaceum*, *F. langsethiae*, *F. poae* and *F. sporotrichioides*. It was found that the differentiation of *F. poae* from the other three species could be achieved with excellent performance. Differentiation between the other species was more difficult. The overall classification metric between four sample categories reached 70% with the Support Vector Machine model calculated in a cross-validation scheme.

It has been shown that the features extracted from the transient response of the sensor to the perturbation caused by the modulation of the temperature of the sensor heater resulted in better classification performance than when the model was built with the features extracted from the response of the sensor in the gas adsorption phase. By fusing the features from both phases of the sensor response, we were able to achieve an additional improvement in classification performance. Therefore, we can conclude that using both types of sensor responses is the better approach.

## Figures and Tables

**Table 1:** List of sensors of the electronic nose and their target gases and odors [9].

Sensor	Target Detection
TGS 2600	Highly sensitive to low concentrations of gaseous air pollutants such as hydrogen and carbon monoxide, for example in cigarette smoke. Can detect hydrogen in a concentration of several ppm.
TGS 2602	Highly sensitive for low concentrations of odor-intensive gases such as ammonia and H_2_S originating from waste materials in office and residential environments. Very sensitive to low concentrations of VOCs such as toluene from wood surfaces and building products
TGS 2603 *	Very sensitive to low concentrations of odorous gases such as odors from the amine range and sulfurous odors from waste materials or spoiled food such as fish.
TGS 2610	Uses filter material that eliminates the influence of interfering gases such as alcohol and is highly selective for LP gas.
TGS 2611	Uses filter material that eliminates the influence of interfering gases such as alcohol and is highly selective for methane gas.
TGS 2620	Has a high sensitivity for organic solvents and other volatile vapors and is therefore suitable for organic vapor detectors/alarms.

* Finally, the signals from the TGS 2603 sensor were not used for analysis due to much longer sensor relaxation time. We observed that the subsequent sensor measurements’ responses were influenced by the previous ones.

**Table 2:** Components used in the constructed electronic nose device with the corresponding cost. The prices can change significantly if lower or higher quality parts are used.

TGS 2600	EUR 25
TGS 2602	EUR 25
TGS 2603	EUR 25
TGS 2610	EUR 25
TGS 2611	EUR 25
TGS 2620	EUR 25
Electronic parts (processor, multiplexer, DAC, and other parts)	EUR 25
Sensor chamber design	20 h
Sensor chamber 3D print	EUR 5
Pneumatic pump and accessories (vanes, locks)	EUR 150
Control unit package	EUR 20
Electrical circuit assembly	70 h
Setup assembly	5 h
Control software	80 h
Total	EUR 445
	175 h

**Table 3:** Description of the features extracted from the sensor response curves (Figure 2).

Adsorption phase of the response	
min-A	Minimal value of the response. This feature was extracted only for the TGS-2602 sensor, since other sensors’ minimum response was observed at the start of the adsorption phase.
ran-A	Range between the final state of the response and the minimum response.
auc-A	Area under the sensor response curve, which is equivalent to the average response of the sensor measured during that phase of the response.
slb-A	Slope of the response curve at the beginning of the adsorption phase, measured after the response reached the minimum value.
sle-A	Slope of the response curve at the end of the adsorption phase.
Temperature modulation phase of the response	
min-T	Minimal value of the response reached after sensor heater temperature drop.
ran-T	Range between the final state of the response and the minimum response.
auc1-T	Area under the response curve, from the start of the modulation phase, until reaching of the minimum of the response.
auc2-T	Area under the response curve, from the moment of minimal response, until the end of observation.
slb-T	Slope of the response curve, just after the start of the temperature modulation phase.
slm-T	Slope of the response after the minimum of the response (average over 50 s after the minimum).

**Table 4:** Measures of the performance of classification models obtained by Logistic Regression and Support Vector Machine algorithms estimated with the leave-one-out cross-validation method. Comparison of different sets of predictors extracted from the adsorption phase (A), the temperature modulation phase (T), both phases of the sensor response (AT). For Precision and Recall, the average of the performance for all species is compared with the performance of *F. poae* alone. LCL and RCL denote 95% left and right confidence intervals limits, respectively, calculated using the bootstrap technique.

		A			T			AT		
		Value [%]	LCL	UCL	Value [%]	LCL	UCL	Value [%]	LCL	UCL
Log.Reg.	Accuracy	54.4	38.6	77.2	57.9	47.4	84.2	63.2	59.6	86.0
	Precision	50.5	33.7	68.4	57.0	50.3	82.6	62.9	54.6	85.0
	Precision (*F. poae*)	87.5	66.7	100.0	92.9	75.0	100.0	93.3	75.0	100.0
	Recall	54.2	35.6	74.3	58.4	49.0	82.2	63.4	50.3	84.8
	Recall (*F. poae*)	100.0	40.0	100.0	92.9	60.0	100.0	100.0	40.0	100.0
SVC	Accuracy	45.6	36.8	78.9	61.4	49.1	86.0	70.2	49.1	89.5
	Precision	46.0	36.1	52.4	58.2	49.4	84.5	70.1	43.1	89.6
	Precision (*F. poae*)	93.3	75.0	100.0	93.3	75.0	100.0	93.3	75.0	100.0
	Recall	45.6	35.5	76.7	62.1	41.3	85.3	70.4	43.1	88.6
	Recall (*F. poae*)	100.0	60.0	100.0	100.0	71.4	100.0	100.0	60.0	100.0

**Figure A1:**
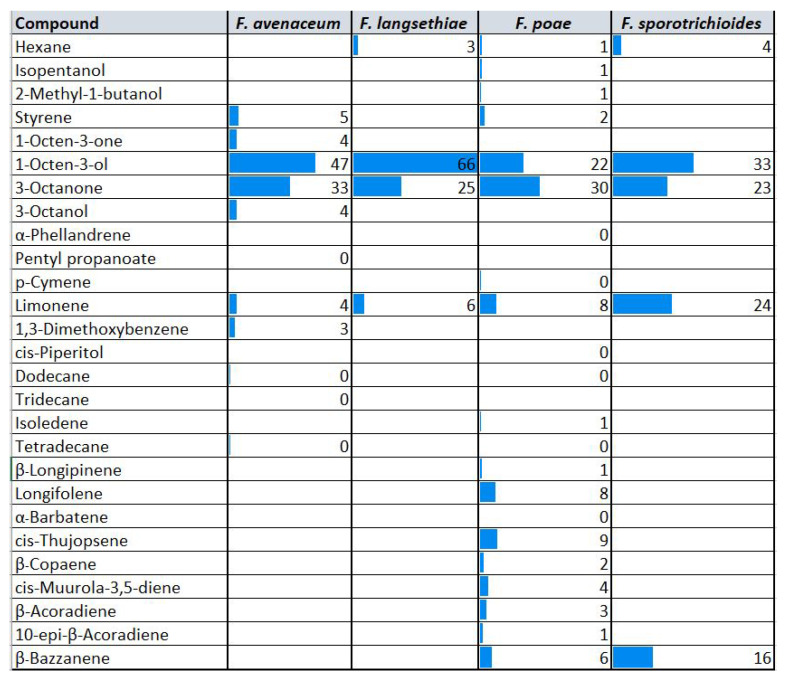
Chemical compounds detected by the GC-MS analysis [56]. The numbers (rounded to whole percentages) indicate the percent of the total ion current collected during the measurement of a sample. The bars are plotted to facilitate visual comparison of data. Empty cells in the table indicate that the component has not been detected, and zero indicates that the found percentage of the detected compounds in the sample was below one percent.

## Data Availability

The original contributions presented in the study are included in the article; further inquiries can be directed to the authors. The raw data supporting the conclusions of this article will be made available by the authors on request.

## Appendix A. GC-MS Measurement of Volatiles

In another experiment [56], we performed GC-MS analysis of the samples of wheat grain infested by the four studied species of *Fusarium* fungi. The measurements were performed using samples other than those used in the electronic nose measurement but prepared using the same procedure. We believe that these results may be helpful for the interpretation of the results obtained by the electronic nose measurements presented in this manuscript.

The presented data does not demonstrate the absolute magnitude of the concentration of a chemical component found in the sample, but the proportions of the chemical components detected in the measured sample (Figure A1).

The main differences between the studied sample categories, instead of an analysis of the whole list of detected chemical compounds, can be visualized after the reduction of dimensionality by transformation by the Principal Components Analysis method (Figure A2).
